# Assessment of Selected Heavy Metals Concentration Level of Drinking Water in Gazer Town and Selected Kebele, South Ari District, Southern Ethiopia

**DOI:** 10.1155/2023/1524850

**Published:** 2023-05-03

**Authors:** Woldesenbet Bafe Dilebo, Misganew Desta Anchiso, Tsirsit Tereke kidane, Meselu Eskezia Ayalew

**Affiliations:** ^1^Jinka University, Department of Chemistry, Jinka, Ethiopia; ^2^Oda Bultum University, Department of Chemistry, Chiro, Ethiopia

## Abstract

Drinking water quality is fundamental to human physiology and health. The aim of this study was to assess the quality of drinking water in Gazer Town and selected kebele, in south Ari district, South Omo zone, Southern Ethiopia. A total of four drinking water samples were collected from densely populated urban areas of the Gazer Town and one rural Kebele. All the collected samples were analyzed for eight heavy metals, (Cd, Co, Cu, Cr, Fe, Mn, Pb, and Zn) using standard procedures. The results were compared with other national and international standards. Among the analyzed samples, drinking water samples collected from selected kebele (Aynalem kebele), show mean concentrations heavy metals in (*μ*g/L), (Mn (973 ± 10), Cu (1068 ± 1.5), Cr (2785 ± 25), Fe (4302 ± 15), Cd (1218 ± 18), Pb (720 ± 12), Co (1478 ± 3), and Zn (1790 ± 5)), and the results revel except, Co and Zn metals, all show concentrations higher than the national and international standards (such as USEPA (2008), WHO (2011), and New Zealand) recommended values. Among the eight heavy metals analyzed from drinking waters in Gazer Town, Cd and Cr were below the method detection than that of all sampling area. However, the concentration of Mn, Pb, Co, Cu, Fe, and Zn were ranged from mean values of 9 *μ*g/L, 17.6 *μ*g/L, 7.6 *μ*g/L, 12 *μ*g/L, 765 *μ*g/L, and 494 *μ*g/L, respectively. Except Pb metals, the analyzed metals in waters were below the currently recommended guidelines for drinking. Therefore, the government should adopt some treatment technologies such as sedimentation and aeration to minimize the concentration of zinc for safe drinking the water to the community of Gazer Town.

## 1. Introduction

Water is extremely important nutrient for the human body and processing of various materials in industries. Even though, living organisms cannot exist without water and almost all industries require water to operate. As a result, water is important for life [1, 2]. In addition to that safe drinking water is a human birthright, as much a birthright as clean air. As a matter of fact, in most of the African and Asian countries, even in relatively advanced countries such as India, safe drinking water is not easily available because of contaminant issues. Currently, more than one billion peoples have lack of access to safe drinking water and in addition to that around 2.5 billion do not have access to adequate sanitation services [3]. This is due to smaller scale water quality assessments [4]. Therefore, assessing of safe drinking water is mandatory for human being to survive in life. Nowadays, drinking water is obtained from a variety of water sources such as wells, rivers, lakes, reservoirs, ponds, and ground water. Especially, more than half the world's population depends on groundwater for survival because of it is an important source for drinking purpose and more reliable than the other water source [1, 5, 6]. However, water source is a great risk to human health once it is contaminated. When water becomes contaminated with toxic compounds, it must be treated before human consumption. Similarly, contaminated water can be dangerous to plants and animals when their metabolic processes are disrupted by drinking from these living things [7].

Contaminations of water occur because of a few key factors, i.e., industrial and sewage effluent discharge, agricultural industry, illegal garbage disposal, and leakage of leachate from landfills [8]. All these, water pollutant mainly consist of heavy metals, microorganisms, fertilizers, and thousands of toxic organic compounds [9]. Especially, disposal of heavy metals containing wastewater is a major environmental issue since contaminants can ultimately gain access to surface and groundwater [1] and also gain to human trough by using of these water sources for drinking purpose. Consequently, these cause a number of water borne diseases as the causes of health hazards [10]. Some heavy metals are essential for the health of living organisms. However, if the concentration level of metals is higher than that of recommended limits, their roles immediately are changed to a negative dimension and some of them are harmful even in small amount [10–12].

The level of seriousness of the problem is much worse in developing countries, especially in rural area. Mainly because of lack of technology, awareness and economic deficiency to treat metals from their drinking water, this results the maximum concentration limit (MCL) of metals from proposed WHO limits [13–15]. This enforces to take a great attention to protect the quality of drinking water. Therefore, monitoring a wider range of water quality parameters and other indicators related to drinking water services and their impacts are essential to provide a nuanced understanding of the risk factors for contamination of different settings and drinking water services, including those used by vulnerable populations [4].

Currently, the level of heavy metal concentration in water sample has been determined by using advanced spectroscopic methods such as atomic absorption spectrometry (AAS), flame atomic absorption spectrometry (FAAS), graphite furnace atomic absorption spectrometry (GFAAS), microwave plasma atomic emission spectroscopy, and inductively coupled plasma spectroscopy (ICPS) [16].

Basically, studies and research findings are important to contribute some valid knowledge to the wellbeing of the society and for the advancements of society about the quality of water. The aim of this study was to determine the concentration level of some selected heavy metals of drinking water in Gazer Town and selected kebele South Ari district, South Omo Zone, Southern Ethiopia. According to reviews literatures, there were no further studies have been conducted in Gazer Town, on the level of heavy metal concentration regarding quality drinking water; and, the groundwater that are found on one selected kebele (such as Aynalem) is not used for drinking purposes because of unpleasant taste, odor, and color. Therefore, all this also might facilitate the researcher to conduct these studies regarding to concentration level of heavy metals in these areas from the point of view of water quality parameters. Also, the researcher believes that this small study will be a step stone and contribute its part for those people who are interested in the further study of this topic. Inspired by the motivations mentioned above, we used advanced spectroscopic techniques such as inductively coupled plasma optical emission spectroscopy (ICP-OES) to determine the concentration level of some heavy metals such as (Cd, Co, Cu, Cr, Fe, Mn, Pb, and Zn) of drinking water in Gazer Town and one selected kebele South Ari district, South Omo Zone, Southern Ethiopia and compare the results with other international standard agency. Results revel that, except Co and Zn metals the existence of high concentration of other metals compared with standard agency in selected kebele. In addition to that, some heavy metals concentration level is below detection limits like (Cd and Cr) in Gazer Town.

### 1.1. Description of the Study Area

**Figure 1:**
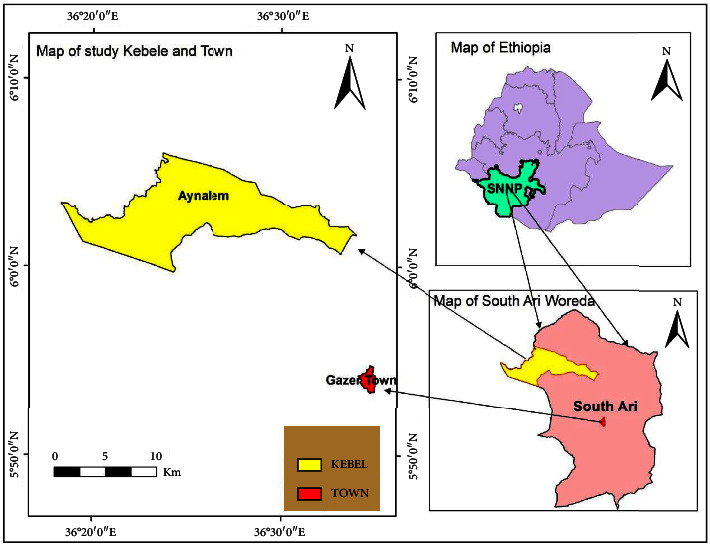
Map of study area (from GIS).

Water samples were collected South Omo Zone, Southern Ethiopia specifically Gazer Town shown on the map (Figure 1). It is the nearest to the capital of the zone Town Jinka and 17 km away from it. Geographically, the area lies between 13° 40′ N and 14° 27′ N and between 36° 27′ E and 37° 32′ E. The traditional agro ecologies of the Woreda are Dega (30%), Woina-dega (65%), and Kola (5%) of the total areas. The Woreda has bimodal type of rain fall pattern and the mean annual rainfall ranges between 601–1600 mm. The mean annual temperature ranges between 10°C–1°C. Based on the 2007 national census conducted by the central statistical agency of Ethiopia, the Woreda has a total total population of 168,225, which is about 35% of the zone population [17].

## 2. Materials and Methods

### 2.1. Instrumentation and Apparatus

Polyethylene bottles and polyethylene bags were used to collect groundwater samples. Borosilicate Erlenmeyer flask and hot plate were used to digest the collected water samples. The pipettes (5 mL), 100 mL, 50 mL volumetric flask, 50 mL of Erlenmeyer flask and beakers were used to dilute the standards and samples solution. Inductively coupled plasma-optical emission spectrophotometer (ICP-OES), (Perkin Elmer MODEL Optima 8000, U.S.A.) equipped with Argon gas, with Plasma, auxiliary, Nebulizer, and RF Power, for the determination of Manganese, Iron, Cobalt, Zinc, Copper, Cadmium, Lead, and Chromium were used.

### 2.2. Chemicals and Standard Solutions

Chemicals that were used in the analysis are analytical grades. 35.4% of HCl (Loba, chemical Ltd, laboratory reagent, India) and 69% (Analytical *R*, IMO: Nitric acid solution BDH Laboratory supplies, England) of HNO_3_ were used to digest the water samples. Stock standard solution of the metals Cu (1000 mg/L), Fe (1000 mg/L), Mn (1000 mg/L), Cd (1000 mg/L), Zn (1000 mg/L), Pb (1000 mg/L), Cr (1000 mg/L), and Co (1000 mg/L) prepared for inductively coupled plasma-optical emission spectrophotometer (ICP-OES), (Perkin Elmer MODEL Optima 8000, U.S.A.) were used for the preparation of calibration curves for the determination of metals in the sample. Distilled water was used for cleaning of glassware and dilution of sample solutions.

### 2.3. Water Sampling and Transportation

Representative samples of drinking water (spring and ground) were collected from Water samples were collected from three sampling sites Gazer town, on May 2019. Grab sampling technique was employed on selecting the site of the study area. Water samples were collected in polyethylene bottles from three different sampling sites (one from catchment tankers and two from along the three distribution network reservoirs) by using polyethylene glass [18]. The samples were transported to the laboratory and stored at 4°C until analysis.

### 2.4. Digestion Sample Water

Three replicates of 100 mL of water samples were taken into 50 mL of Erlenmeyer flask from each ground water and acidified with 3 : 1 HNO_3_/HCl ratio. Then, the samples were heated on a hot plate to reduce the volume to a defined level (25 mL). Finally, the remaining part of the samples were cooled and (filtered through Whatman paper filter paper). Finally, samples were diluted to 100 mL volumetric flasks and ready for analysis by Inductively Coupled plasma-optical Emission spectrophotometer.

### 2.5. Calibration Standard Solutions

The calibration standard solutions were used to calibrate the instrument response with respect to the analyte concentration. Standard solutions of eight points were prepared for each analyte from their respective working standard solutions (100 mg/L) for metals. The calibration standard concentrations were within the working linear range of the instrument used for analysis. The prepared calibration standards: standard 1 (*S*_1_), standard 2 (*S*_2_), standard 3 (*S*_3_), standard 4 (*S*_4_), 5 (*S*_5_), 6 (*S*_6_), 7 (*S*_7_), and standard 8 (*S*_3_) for each analyte are given in Table 1.

### 2.6. Instrumental Technique and Optimal Condition of ICP-OES

Concentrations of the heavy metals in all samples were measured using Inductively coupled plasma-optical emission spectrophotometer (ICP-OES). To analyze the sample on the ICP-OES, the instrument was adjusted in appropriate manner as shown in Table 2 such as, wavelength selector; plasma, auxiliary, Nebulizer, RF Power, gas, and optimal values of these parameters are those, which yield maximum absorbance value.

### 2.7. Data Analysis

The heavy metals were compared with WHO and Ethiopian Standard Agency (ESA) guidelines for drinking water which was reported in Table 3. The obtained data were also treated with one-way ANOVA to assess the variations of the parameters among the spring water samples and ground samples analyzed by using Origin pro 2019 version software.

## 3. Results and Discussion

### 3.1. Calibration Curve and the Determination Metal Concentrations

**Figure 2:**
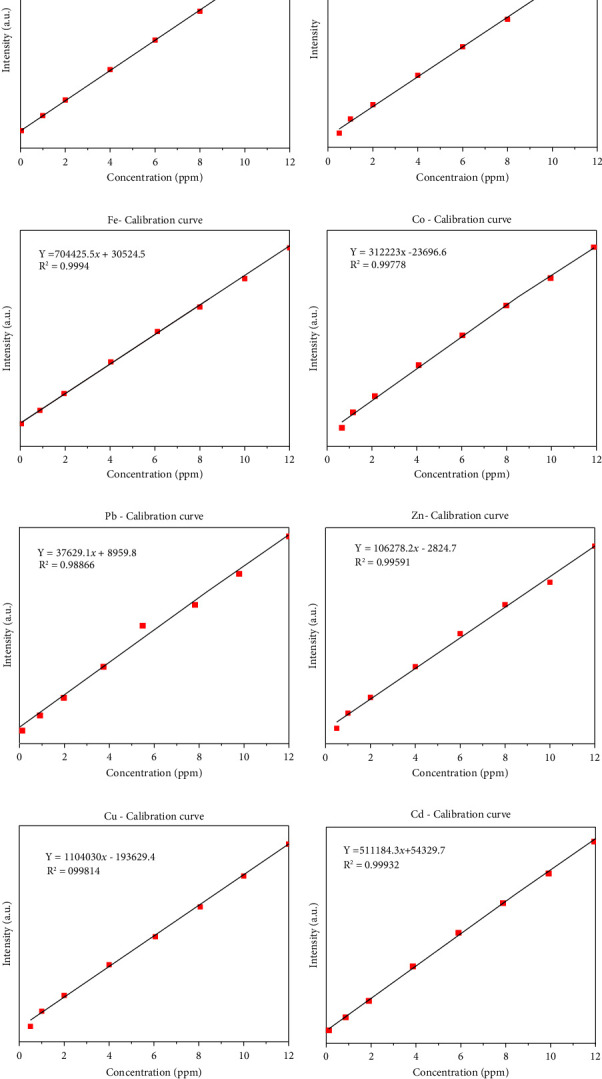
Calibration graphs for standard solution.

The quality of data resulted from ICP-OES measurements are affected by sample digestion, calibration, and standard solution preparation procedures. In this study, the instrument was calibrated by using eight series of working standard solutions, prepared from intermediate solutions for each metal under determination. The analytical calibration curves were plotted by using prepared standard solutions for every element (Cd, Cr, Pb, Zn, Cu, Fe, Co, and Mn). The value of correlation coefficients of calibration curves for each toxic and essential metal as well as the linearity of data on the calibration for Cr, Mn, Fe, Co, Pb, Zn, Cu, and Cd, are seen in Figures 2(a)–2(h).

### 3.2. The Concentration of Heavy Metal in Drinking Water

In this study, all analyzed heavy metals (Cd, Co, Cr, Cu, Fe, Mn, Pb, and Zn) were detected in the water samples, and their concentrations (mean ± SD) are presented in Table 4.

**Figure 3:**
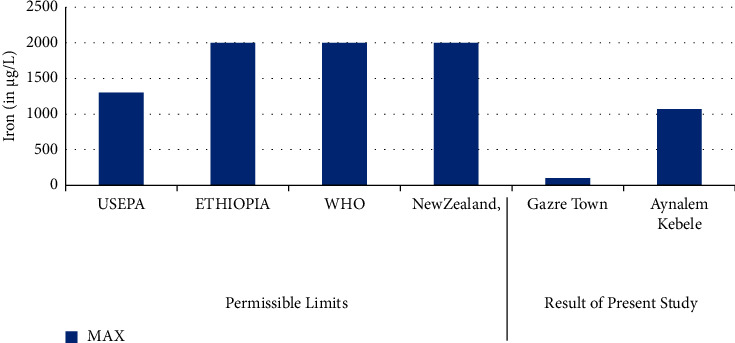
Comparison of iron of the present study with permissible limits set by different agencies.

In Figure 3, the lowest concentration of iron was detected in Gazer Town sample site while the highest concentration of iron was detected in Aynalem Kebele water site. All samples analyzed were below WHO permissible limits for iron in drinking water of 3,000 *μ*gL^−l^ [19].

**Figure 4:**
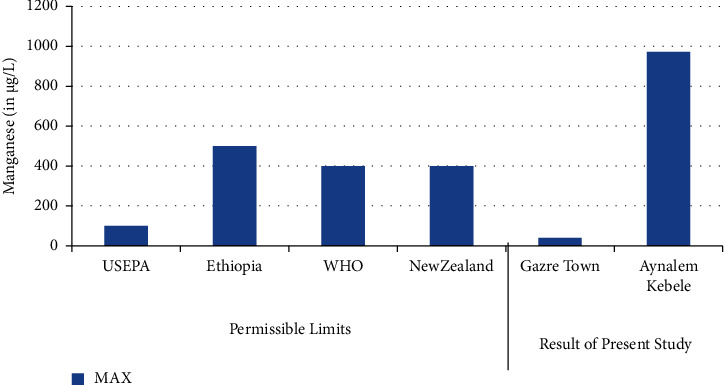
Comparison of manganese of the present study with permissible limits set by different agencies.

The lowest concentration of manganese was found in Gazer town water site while the highest concentration was observed in Aynalen Kebele water site as shown in (Figure 4). The water samples analyzed in Gazer town were below WHO permissible limits but the water samples analyzed at Aynalem Kebele were above WHO permissible limits for manganese in drinking of 400 *μ*gL^−l^ (WHO 2017).

**Figure 5:**
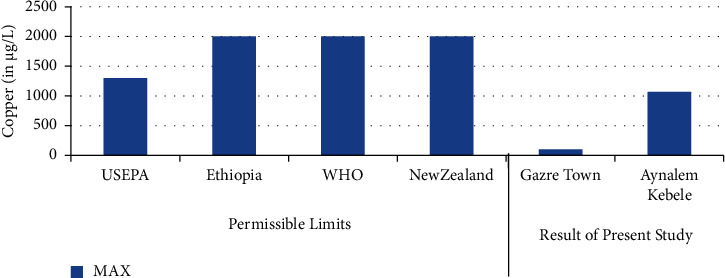
Comparison of copper of the present study with permissible limits set by different agencies.

The highest concentration of copper was in Aynalem Kebele water sample and the lowest concentrations were observed at Gazer town sample site as shown in (Figure 5). All the water samples analyzed for copper were found below WHO permissible limits of 50 *μ*gL^−l^ [19].

**Figure 6:**
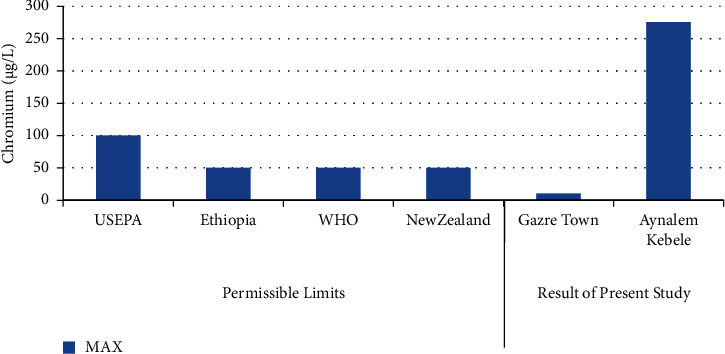
Comparison of chromium of the present study with permissible limits set by different agencies.

In Figure 6 shows the highest concentration of chromium was record in Aynalem kebele samples site and the lowest concentration were record in Gazer town kebele. The Aynalem kebele samples site of that chromium was detected are above the WHO maximum permissible limits and Gazer town sample site of that chromium was detected below WHO maximum permissible of 50 *μ*gL^−l^ [19].

**Figure 7:**
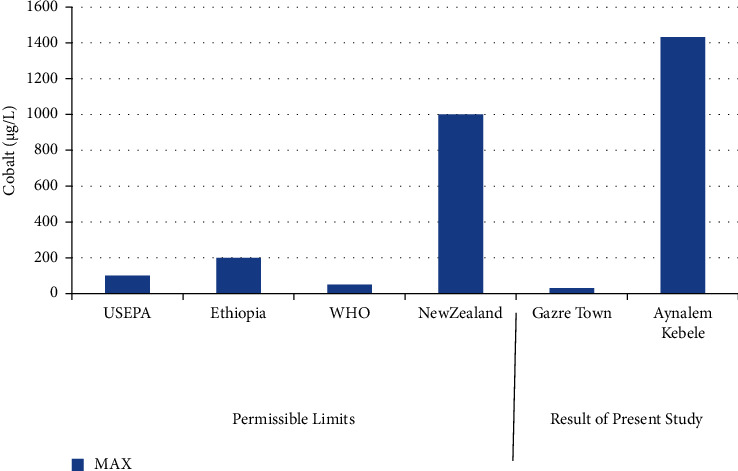
Comparison of cobalt of the present study with permissible limits set by different agencies.

The highest concentration of cobalt was record at Aynalem Kebele water sample and the lowest concentrations were observed at Gazer town sample site shown in (Figure 7). The Aynalem Kebele water samples analyzed for cobalt were found above WHO permissible limits and Gazer sample site were found below WHO permissible limits of 50 *μ*gL^−l^ (WHO 2011) [20].

**Figure 8:**
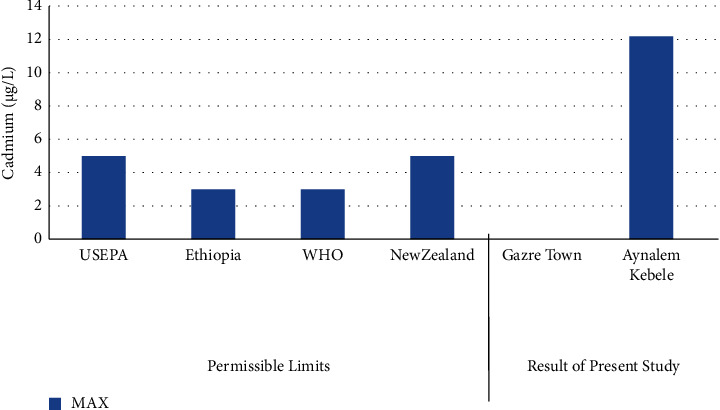
Comparison of cadmium of the present study with permissible limits set by different agencies.

Also as shown in Figure 8, cadmium was detected only in Aynalem Kebele sample site. It was not detected in Gazer water sample site. The Cadmium detected in Aynalem Kebele sample site was above WHO permissible limits for in Cadmiuin drinking water of 50 *μ*gL^−l^ [20].

**Figure 9:**
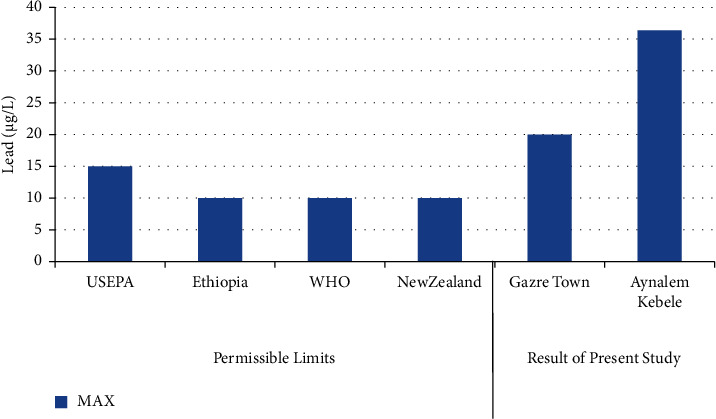
Comparison of lead of the present study with permissible limits set by different agencies.

The highest concentrations of lead in were obtained in Aynalem Kebele water samples however, the lowest concentrations were found at Gazer town water sample site in (Figure 9). Therefore, the lead detected in all sample sites is above WHO permissible limits in drinking water of 10 *μ*gL^–l^ [20].

**Figure 10:**
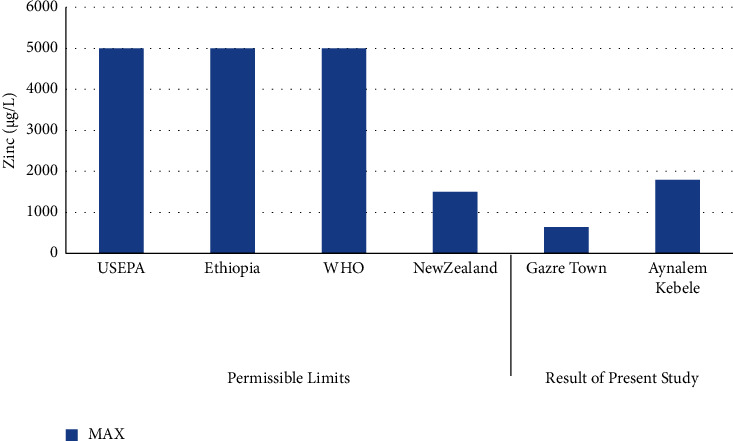
Comparison of zinc of the present study with permissible limits set by different agencies.

The least concentration of zinc was observed in Gazer Town water sample site while the highest concentration was recorded in Aynalem kebele water sample site shown in (Figure 10). All the water samples collected from Gazer Town contains zinc metals below the WHO maximum permissible limit of 5,000 *μ*gL^–l^ (WHO 2017).

### 3.3. Lead Status

Lead occurs mostly in association with zinc and gets into water from corrosion of zinc coated (“galvanized”) pipes [21] and leaching from water distribution pipes [22, 23]. The concentration of Pb obtained from Aynalem kebele and Gazer town site water sample site were around (720 ± 12 *μ*g/L) and 13 ± 1.4 *μ*g/L respectively shown in Table 4. Therefore, the obtained result shows that high concentration of Pb in both sampling area beyond the maximum permissible limit given by WHO (2004) and USEPA (2011). As results this may case health problem to the society unless strict measurement will be taken.

### 3.4. Cadmium and Chromium

Mostly, high concentration of cadmium metal related with industrial and mining activities in the sampling areas [22, 23] and chromium metal related with Soil leaching. Especially, the high concentration of cadmium in drinking water is mostly related with corrosion of metal pipes, water tanks, and plumbing systems [24, 25]; this causes a disease of like short periods of time: nausea, vomiting, diarrhea, muscle cramps, salivation, sensory disturbances, liver injury, convulsions, shock, and renal failure as reported [26].

Our results reveal that the concentration of cadmium and chromium metals in Gazer town water sampling area was found to be below the detection limit. Therefore, no health problem related with these metals. However, high concentrations were observed in Aynalem kebele water sampling area regarding from maximum admissible limit of international organizations like, WHO (2017) reported in (Figures 6 and 8) and in table.

### 3.5. Iron

Iron is the fourth most abundant element by mass in the earth's crust. In water, it occurs mainly in ferrous or ferric state. Iron in surface water generally present is ferric state [27]. As shown in Table 5, the concentration of Fe in the study area ranged from 4302 ± 15 *μ*g/L in Aynalem kebele sample water to 677 ± 7 *μ*g/L in Gazer Town sampling site. The results were found above the maximum permissible limit (0.3 mg/L) set by WHO (2004). This may cause health problem related with iron in the study area. But in the studied areas of Gazer town, iron content consistent with the desirable concentration limit of drinking water set by WHO (2011) and other international organizations, whereas high concentration was observed in the sampling area of Aynalem kebele. As reported in [24, 28], the prolonged consumption of drinking water with high concentration of iron may lead to liver disease called hemosiderosis.

### 3.6. Manganese

Manganese one of the most important elements used for the proper functioning of both humans and animals, as it is required for the functioning of many cellular enzymes. Nevertheless, high levels of manganese may harm brain development in infants and young children [25, 29]. Similarly as shown, the concentration of manganese was recorded 973 ± 10 *μ*g/L at Aynalem kebele and 41 ± 0.00 *μ*g/L in gazer town sample site. The result obtained from sites were below the permissible limit recommended by international water quality standards like WHO (0.1 *μ*g/L) and USEPA (0.5 *μ*g/L) guide lines. So, these sites were safe for domestic and irrigational use. In this study, manganese concentration which recorded a water sample from Gazer town is with complying the maximum admissible limit set by international agencies. But high concentration manganese was observed in the sampling site of Aynalem kebele.

### 3.7. Zinc

The dissolved concentration of zinc in the water samples from Aynalem kebele site recorded 1790 ± 5 *μ*g/L and 644 ± 1.9 *μ*g/L recorded at gazer town water sample. As shown in Table 5, the mean concentrations of zinc metal in the three sample sites were recorded below the permissible limit recommended by WHO and USEPA guideline range (3 mg/L) and (5 mg/L), respectively.

### 3.8. Copper

Copper is Exposure to high doses of copper can cause health problems. Short-term exposure to high levels of copper can cause gastrointestinal distress, nausea, vomiting, and diarrhea [26, 30]. Also, high concentration of cobalt in drinking water can cause interstitial lung disease. They are mostly present naturally in rock, soil, water, plants, animals, and air. In present study, the concentration of zinc and copper in both drinking water sampling sites of (Gazer town and Aynalem) were, below the WHO and different national guidelines for drinking water quality (Table 5). Therefore, from above-given points of view, no adverse effects concerning with this element in water for drinking purposes. Also, the concentration of Cobalt in all sampling area is within WHO (2017) maximum recommended range.

## 4. Conclusion and Remarks

The main goal of this paper was to assess the status of drinking water quality in Gazer town and selected kebele (Aynalem) areas regarding concentration level heavy metals. Most of the water samples are colorless and odorless. However, some water samples collected from Aynalem kebele are slightly colored due to muddiness. The current study results showed that the concentrations of most heavy metals in the water samples were within the permissible limit of the World Health Organization and Ethiopian Standard guidelines for drinking water quality, and except lead (Pb), all analyzed heavy metals in Gazer town were below the established guideline values. This high concentration of lead was observed due to corrosion of zinc coated (“galvanized”) pipes or leaching from water distribution pipes and geogenic contaminants. Therefore, no health and aesthetic problems concern the analyzed heavy metals of the waters for drinking. But the results show that drinking water from Aynalem kebele is not good regarding these selected heavy metal concentration levels, because it is higher than the MAL set by WHO, 2017 and different national guidelines for drinking water quality. This is an indication of weak drinking water treatment practices in these areas which, in turn, have implications on the health of the people.

## Figures and Tables

**Table 1:** Series of working standards for determination of trace metals in water sample.

Analytes	Calibration standard solutions (mg/L)							
	*S*_1_	*S*_2_	*S*_3_	*S*_4_	*S*_5_	*S*_6_	*S*_7_	*S*_8_
Mn	0.5	1	2	4	6	8	10	12
Fe	0.5	1	2	4	6	8	10	12
Cr	0.5	1	2	4	6	8	10	12
Co	0.5	1	2	4	6	8	10	12
Zn	0.5	1	2	4	6	8	10	12
Ni	0.5	1	2	4	6	8	10	12
Cu	0.5	1	2	4	6	8	10	12
Pb	0.5	1	2	4	6	8	10	12
Cd	0.5	1	2	4	6	8	10	12

**Table 2:** Instrumental operating conditions used for flame atomic absorption spectrometer.

Elements	Wave length in (nm)	Plasma	Auxiliary	Nebulizer	RF power	Gas
Mn	279.5	10	0.3	0.7	1300	Ar
Zn	213.9	10	0.3	0.7	1300	Ar
Fe	248.3	10	0.3	0.7	1300	Ar
Cd	368.4	10	0.3	0.7	1300	Ar
Cu	324.7	10	0.3	0.7	1300	Ar
Pb	283.2	10	0.3	0.7	1300	Ar
Cr	357.5	10	0.3	0.7	1300	Ar
Co	228.6	10	0.3	0.7	1300	Ar

**Table 3:** Drinking water contaminants and maximum admissible limit set by different national and international organizations (for health risk and aesthetic value) [3].

	Heavy metals (*μ*g/L)									
	As	Cd	Co	Cr	Cu	Fe	Mn	Ni	Pb	Zn
USEPA, 2008	10	5	100	100	1300	300	50	100	15	5000
EU, 1998	10	5	NM	50	2000	200	50	20	10	NM
WHO, 2008	10	3	NM	50	2000	NGL^*∗∗∗*^	400	70	10	NGL^*∗∗*^
Iranian, 1997	50	10	NM	50	1000	1000	500	NM	50	NM
Australi, 1996	7	2	NM	50^c^	2000	300^c^	500	20	10	3000^b^
Indian, 2005	50	10	NM	50^c^	1500	300	100	20	100	5000
New Zealand, 2008	10	4	1000	50	2000	200	400	80	10	1500

^*∗*^NM = not mentioned; ^*∗∗*^NGL = no guideline, because it occurs in drinking water at concentrations well below those at which toxic effects may occur; ^*∗∗∗*^no guideline, because it is not of health concern at concentrations normally observed in drinking water but may affect the acceptability of water at concentration above 300 *μ*g/L, ^b^based on quality (aesthetic) not safety (health risk), ^c^chromium as Cr^+6^ not total Cr.

**Table 4:** Mean ± SD (in *μ*g/L) of major heavy metals Pb, Cr, Mn, Cd, Co, Zn, and Cu drinking water samples.

Metals			Mn	Zn	Co	Cu	Cr	Fe	Cd	Pb
Places	Gazer town	*S*_1_	4 ± 0.00	644^*∗*^ ± 1.9	1^*∗*^ ± 1	4^*∗*^ ± 0.2	BDL	677^*∗*^ ± 7	BDL	13 ± 1.4
		*S*_2_	BDL	BDL	20^*∗*^ ± 2	30^*∗*^ ± 2	BDL	BDL	BDL	20 ± 0.7
		*S*_3_	14 ± 0.0	344 ± 09	2 ± 0.1	2 ± 0.32	1 ± 0.2	427 ± 2.3	BDL	20 ± 0.2
	Aynalem Kebele	*S*_4_	973 ± 10	1790 ± 5	1478 ± 3	1068 ± 1.5	2785 ± 25	4302 ± 15	1218 ± 18	720 ± 12

^*∗*^ND = not detected.

**Table 5:** Compression of studied samples with different guidelines for drinking water quality (for health risk and aesthetic value) set by different national and international organizations.

Elements	Tested method	Standard requirement by different organization				Condition of studied samples Gazer town	Condition of studied samples Aynalem Kebele
		Max (*μ*g/L)					
		Ethiopia, 2011	USEPA, 2008	WHO, 2011	NewZealand, 2008		
Cu	ICP-OES	2000	1300	2000	2000	Safe	Safe
Cd	ICP-OES	3	5	3	4	Safe	Not safe
Cr	ICP-OES	50	100	50	50	Safe	Not safe
Zn	ICP-OES	5000	5000	5000	1500	Safe	Safe
Mn	ICP-OES	500	50	400	400	Safe	Not safe
Fe	ICP-OES	0.3	300	300	200	Safe	Not safe
Co	ICP-OES	200	100	20	1000	Safe	Safe
Pb	ICP-OES	15	15	10	10	Not safe	Not safe

Source for international standards and Mebrahtuet al., (2011) [3].

## Data Availability

(1) All data used to support the findings of this article are expressed (found) within the article or the main manuscript. (2) The methodology is found in the references that means the journal cited in the main document.
